# Isolation and identification of peste des petits ruminants virus from goats in Egyptian governorates

**DOI:** 10.14202/vetworld.2021.926-932

**Published:** 2021-04-17

**Authors:** Sahar Ahmed, Wafaa Abd El Wahab Hosny, Mervat Mahmoud, Mohammed Abd El-Fatah Mahmoud

**Affiliations:** 1 Department of Cell Biology, Genetic Engineering and Biotechnology Research Dvision, National Research Centre, 12622 Dokki, Giza, Egypt; 2 ELISA Unit and Virus Strains Bank, Animal Health Research Institute, Agriculture Research Centre, Dokki, Giza, Egypt; 3 Department of Parasitology and Animal Diseases, Veterinary Research Division, National Research Centre. Dokki Giza, Egypt

**Keywords:** molecular virology, peste des petits ruminants, serological study, small ruminants

## Abstract

**Background and Aim:**

The peste des petits ruminants (PPR) is a highly contagious disease of small ruminants which negatively affects animal production and the socioeconomic status of farmers. Peste des petits ruminants virus (PPRV) encodes eight proteins, with the viral fusion protein (F) playing a role in virus virulence and stimulating an effective protective immune response. This study aimed to isolate and complete the identification of PPRV circulating in goats in different Egyptian governorates and perform molecular characterization of the *PPRV F* gene.

**Materials and Methods:**

Samples were collected from unvaccinated animals with clinical signs suggestive of PPR. A total of 256 sera were tested for the detection of PPRV antibodies using a competitive enzyme-linked immunosorbent assay (c-ELISA) kit, while 214 samples of blood buffy coat preparation, animal swabs (nasal, ocular, and saliva), and fecal and tissue samples were tested for the detection of the PPRV antigen using an antigen-capture ELISA kit. Molecular diagnosis, gene cloning, blast analysis, and phylogenetic analysis were performed for the molecular characterization of PPRV.

**Results:**

The seroprevalence results of PPRV antibodies in the tested sera showed a total of 67.9% positive samples. The rates of PPR antigen recorded by the antigen-capture ELISA in the swabs (nasal and ocular) and tissue samples were 44.3%, 46.8%, and 43.5%, respectively, with saliva swabs having the highest rate of PPRV positivity (76.4%) and fecal samples having the lowest (33.3%). Molecular characterization of the PPRV Vero cell culture revealed that the circulating PPRV strain belongs to the IV lineage. Blast analysis of the *PPRV F* gene showed 96.7% identity with the PPRV strain Egypt-2014 fusion protein (F) gene, KT006589.1, differing by 43 single-nucleotide polymorphisms.

**Conclusion:**

The results of this study indicate that the emerging PPRV belongs to the IV lineage among small ruminant animals. The findings also indicate the need for an innovative strategy to control and eliminate this disease based on a regularly administered and effective vaccine, a test to distinguish between infected and vaccinated animals, and the need for further study on the protein structure and *PPRV F* gene expression, which should help us to understand the molecular evolution of the virus and control and eliminate PPR disease.

## Introduction

Peste des petits ruminants (PPR) is a widespread animal disease that mainly afflicts goats and sheep. Peste des petits ruminants virus (PPRV) was first reported in Africa on the Ivory Coast; it is a highly contagious disease of small ruminants that have significant economic impacts. These impacts are due to the high morbidity and mortality rates, in the ranges of 10-90% and 50-90%, respectively. The virus belongs to the genus *Morbillivirus*, which is a single-stranded RNA virus of the family Paramyxoviridae [1]. The most common form of PPR is the acute form, which is characterized by depression, high fever, anorexia, nasal, and ocular discharge, followed by mouth erosive lesions, pneumonia, and severe diarrhea [2].

The viral genome is a linear, non-segmented, negative-sense single-stranded RNA that is about 15,948 nucleotides in length [3]. It encodes eight proteins, six of which are structural: Fusion (F), nucleocapsid (N), phosphoprotein (P), large (L), hemagglutinin (H), and matrix (M) proteins [3]. The virus fusion protein (F gene) enables PPRV to penetrate the cell membrane and enter the cytoplasm by affecting the fusion of the virus and host cell membranes. This phenomenon is also responsible for virus spread from cell to cell without the formation of free viral particles. The fusion protein is reported to be critical for inducing an effective protective humoral immune response [4]. The virus circulating in Africa, the Middle East, and Central to Southeast Asia presents four genetically distinct lineages, three of which (I, II, and III) were first described in Africa, including Guinea, Ivory Coast, Senegal, Mali, Burkina Faso, Ghana, Nigeria, Uganda, and Tanzania, and the fourth one (IV) in Asia [1]. However, the Asian lineage was recently found to have become established in some countries of North Africa and Europe, indicating the continuing spread of the virus [5].

It is assumed that the trade in live animals to Egypt from Ethiopia and Sudan has a role in the spread of this infection into North and East Africa [6]. This is supported by the close relationship between the PPRV IV lineage in Egypt and North Africa and the PPRV lineage initially identified in Sudan [7].

The importance of molecularly characterizing the *PPRV F* gene is related to its role in determining virulence in infected animals [4]. Considering the socioeconomic impact of PPR disease, this study aimed to isolate and identify the PPRV circulating in goats in different Egyptian governorates and molecularly characterize the *PPRV F* gene.

## Materials and Methods

### Ethical approval

The experiments were carried out in accordance with the guidelines laid down by the National Research Centre, Animal Ethics Committee, and in accordance with local laws and regulations.

### Study period and location

The study was conducted during the winter of 2017-2019 on goats from small unorganized local Egyptian breed farms in different Egyptian governorates (Cairo, Giza, Dakahlia, Beni-Suef, Sharkia, Gharbia, Alexandria, and Qena, as shown in Table-1)

**Table-1:** Types of the collected samples in relation to the governorates.

Governorates	Serum	B.C	N.S	Conj. S	S.S	F.S	Tissues
Cairo (El Marg)	30	11	7	7	5	3	4
Giza	68	32	5	3	3	2	3
Alexandria	21	4	4	-	-	-	-
Sharkia	25	2	8	5	3	-	3
Gharbia	24	3	10	4	4	-	4
Dakahlia	23	22	12	11	-	3	5
Beni-Suef	39	-	-	-	-	-	-
Qena	26	6	6	2	2	1	5
Total	256	80	52	32	17	9	24

BC=Blood buffy coat, N.S=Nasal swab, Conj. S=Ocular swab, S.S=Saliva swab, F. S=Fecal swab

### Samples

#### Swabs, tissue, and fecal samples

A total of 214 samples of blood buffy coat preparation (n=80), animal swabs (nasal [n=52], ocular [n=32], and saliva [n=17]), fecal (n=9) and tissue (n=24) samples were obtained from different animals (Table-1) and tested to detect the PPRV antigen using capture enzyme-linked immunosorbent assay (ELISA) kit following the manufacturer’s instructions (ID Vet Screen, France). Tissue samples (from lung, lymph node, liver, and intestine) were taken from dead animals. Tissue and fecal samples were prepared in accordance with the work of Clarke *et al*. [8].

#### Serum samples

A total of 256 whole-blood samples were collected from unvaccinated goats with signs suggestive of clinical PPR, in plain tubes for serum preparation (Table-1). All sera were tested to detect PPRV antibodies using a competitive ELISA kit following the manufacturer’s instructions (ID Screen® PPR Competition, ID Vet, Montpellier, France). The optical density (OD) values were converted to PI using the following formula:

Percentage (PI) PI = 100×(OD sample/OD negative control). The cut-off for seropositivity used was ≤50 percent as recommended by the manufacturer (ID Screen®PPR Competition, ID vet, Montpellier, France).

### Molecular diagnosis and characterization of *PPRV F* gene

#### Extraction of PPRV RNA

Total RNA was extracted from 250 μL of blood buffy coat, nasal, ocular, and saliva samples, and PPRV cultures using TRIZOL (Invitrogen, USA), in accordance with the instructions provided by the manufacturer. The RNA pellets were subsequently dried and kept at −80°C until used for molecular study [9].

### Reverse transcription-polymerase chain reaction (RT-PCR)

This assay allows the detection of the four lineages of PPRV. The RNA pellets were resuspended in 25 mL of RNase-free water. RT and partial amplification of the *PPRV F* gene were performed using a single-tube RT-PCR method with the One-step RT-PCR Kit protocol (SuperScript® III Reverse Transcriptase; Invitrogen), following the manufacturer’s instructions.

Primers of the *PPRV F* gene (5´-AGTACAAAAGATTGCTGATCACAGT-3´ and 5´- GGGTCTCGAAGGCTAGGCCCGAATA-3´) [10] were used to confirm the diagnosis of the PPR-positive serological samples. The reaction conditions were 50°C for 30 min to prepare the cDNA; initial activation at 94°C for 2 min; followed by 35 cycles of denaturation at 94°C for 1 min, annealing at 55°C for 1 min, and extension at 72°C for 1 min; and then final extension at 72°C for 7 min. The RT-PCR products were analyzed by electrophoresis on 2% agarose gel.

### Viral isolation

For virus propagation, the capture ELISA-positive samples were confirmed by RT-PCR and inoculated into confluent monolayer Vero cells (green monkey kidney cells) kindly supplied by AHRI, Dokki, Giza, Egypt. Minimal essential media with L-glutamine without sodium bicarbonate and 10% FCS (Sigma-Aldrich) were used for the culturing. The inoculated cultures were examined daily for evidence of a cytopathic effect (CPE). The CPE produced by PPRV was observed within 5 days post-inoculation in the form of cell rounding and aggregation culminating in the formation of syncytia [8]. Three successive blind passages for each negative sample were made to confirm its negativity. The positive tissue culture isolates were confirmed for the PPRV-positive inoculation by partial amplification of the *PPRV F* gene (RT-PCR). For separation of the virus from the culture, the CPE culture flasks were frozen at −80°C and thawed 3 times, after which the culture medium was centrifuged at 4°C for 15 min (3000 rpm). The supernatant was used for viral genome extraction and molecular characterization of the *PPRV F* gene [11].

### Full-length amplification of F gene by RT-PCR

Primers for the full-length *PPRV F* gene (1641 bp) were designed using Primer 3.0 software (Applied Biosystems, USA). The primer sequences of 5´-ATGACACGGGTCGCAATC-3´ and 5´-CTACAGTGATCTCACGTACGACTT-3´ were used for RT-PCR using a One-step RT-PCR kit (Invitrogen, USA) with a thermal cycling program of 45°C for 30 min to prepare the cDNA; initial activation at 95°C for 15 min; followed by 35 cycles of denaturation at 94°C for 1 min, annealing at 50°C for 1 min, and extension at 72°C for 2 min; and then final extension at 72°C for 10 min. The RT-PCR products were analyzed by electrophoresis on 2% agarose gel. The successfully amplified *PPRV F* gene fragments (1641 bp) were cut from the agarose gel and purified using a purification kit (Invitrogen, USA), following the manufacturer’s instructions.

### Cloning of the *PPRV F* gene in T-easy vector

**Figure-1:**
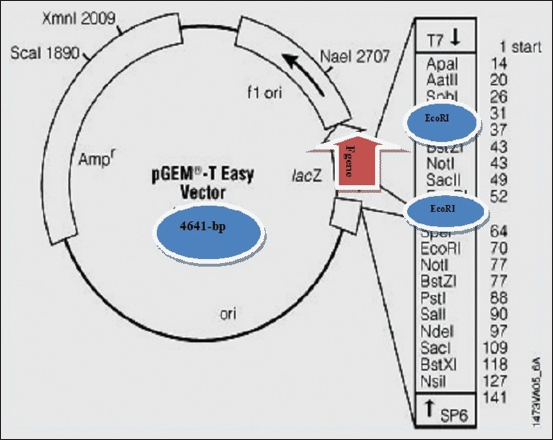
Recombinant of the peste des petits ruminants virus *F*-gene T-easy vector.

The purpose of developing the recombinant *PPRV F* gene was to keep it isolated for molecular characterization of the full-length *PPRV F* gene and for further studies of its protein expression. The ampicillin resistance and white/blue colony selection method were used to select the vector required for cloning. The T-easy vector (3000 bp; Promega Corporation, France) contained an ampicillin resistance gene and white/blue colony selection lacZ indicator were used to select the positive colonies contain the *PPRV F* gene (Figure-1).

The purified full-length *PPRV F* gene (1641 bp) was subjected to cloning into a T-easy vector, following the manufacturer’s instructions. Five microliters of the cloning reaction mixture were added to 50 μL of *Escherichia coli* DH5α competent cells, incubated on ice for 20 min, heat-shocked at 42°C for 45 s, and then put on ice for 2 min. Subsequently, 900 μL of Luria–Bertani (Thermo Fisher Scientific, USA) was added, followed by incubation for about 1.5 h at 37°C. This mixture was distributed in Luria–Bertani agar plates containing 0.5 mM isopropyl-β-D thiogalactopyranoside (Sigma-Aldrich, Germany) and 80 μg/mL X-gal, supplemented with 100 mg/mL ampicillin (Sigma-Aldrich, Germany), and incubated at 37°C.

The transformed bacterial cells contained T-easy vector inserted by PPRV F gene showed ampicillin resistance. The recombinant colonies were white on indicator plates and were selected for new LB agar plates. The white *E. coli* colonies confirming the insertion of the F gene were selected and recultured on LB plates. Purification of the recombinant vector from transformed cells was performed using a plasmid isolation kit and a DNA-spin™ Plasmid DNA Purification Kit (iNtRON Biotechnology, South Korea), following the manufacturer’s instructions.

### Molecular characterization of the cloned *PPRV F* gene

The restriction enzyme *EcoRI* was used following the manufacturer’s instructions to obtain the inserted *PPRV F* gene from the purified recombinant vector. After restriction enzyme digestion of the recombinant vector, the product was analyzed by electrophoresis on 2% agarose gel, and the *PPRV F* gene gel fragment was cut and purified using a purification kit (Invitrogen, USA), following the manufacturer’s instructions. The purified product was used for PCR amplification of the full-length *PPRV F* gene, as previously described. The products successfully amplified by PCR were sent for sequencing to Anses Lab France, French Agency for Food, Environmental, and Occupational Health and Safety. The obtained sequences were subjected to a Blast analysis using the available data in GenBank (https://blast.ncbi.nlm.nih.gov/Blast.cgi). The sequence results were submitted to GenBank.

### Phylogenetic analysis

A phylogenetic tree was constructed based on the *PPRV F* gene for isolates of PPRV (MW039251 Egypt NRC) and available accessions in GenBank. The tree was drawn to scale, with branch lengths representing the number of substitutions per site (next to the branches). The analysis involved 11 nucleotide sequences. The included codon positions were 1^st^+2^nd^+3^rd^+Non-coding. All positions containing gaps and missing data were eliminated. There were a total of 760 positions in the final dataset. Evolutionary analyses were conducted using Molecular Evolutionary Genetics Analysis Version 7.0 for Bigger Datasets (https://pubmed.ncbi.nlm.nih.gov/27004904/) [12].

## Results and Discussion

PPR is one of the most economically devastating diseases affecting sheep and goats in developing countries due to its high morbidity and mortality rates, especially in small flocks [13]. Seroprevalence results of PPR antibodies in the tested goats’ samples from different governorates are presented in Table-2. The results showed variable numbers of PPRV-positive samples among the different Egyptian governorates. The total positive rate was 67.9%. The highest rate was found in Gharbia governorate (83.3%), while the rest of the governorates showed variable rates of PPRV-positive samples. The results of the antibodies in goats’ sera against PPRV between Giza and Beni-Suef governorates confirmed previous findings reported by Mahmoud *et al*. [2] who found that the rates of antibodies in goats sera against PPR were 45.7% at Giza governorate, and 45% at Beni-Suef governorate.

**Table-2:** Seroprevalence of PPRV antibodies detected in goats.

Governorate	No. of samples	+ve samples	+ve %	−ve samples
Cairo (El Marg)	30	18	60	12
Giza	68	40	58.8	28
Alexandria	21	17	80.9	4
Sharkia	25	16	64	9
Gharbia	24	20	83.3	4
Dakahlia	23	18	78.2	5
Beni-Suef	39	29	74.3	10
Qena	26	16	61.5	10
Total	256	174 (67.9%)		82 (32.1%)

The results for PPR antigen determined by immunocapture ELISA in the collected samples showed 105 positive samples (Table-3). The swabs (nasal and ocular) and tissue samples showed rates of positivity for PPR of 44.3%, 46.8%, and 43.5%, respectively. The saliva swabs had the highest rate of PPRV positivity (76.4%), while the fecal samples had the lowest (33.3%). These results are in agreement with those reported by Nafea and Abdallah [14].

**Table-3:** PPRV antigen detected in different goats’ samples.

Governorates	Buffy coat				Nasal swab				Ocular swab				Saliva swab				Fecal swab				Tissues			
					
	N	P	T	% +ve	N	P	T	% +ve	N	P	T	% +ve	N	P	T	% +ve	N	P	T	% +ve	N	P	T	% +ve
Cairo (El Marg)	6	5	11	45	3	4	7	57	3	4	7	57	1	4	5	80	1	2	3	66.6	3	1	4	25
Giza	24	8	32	25	3	2	5	40	1	2	3	66.6	1	2	3	66.6	2	-	2	0	1	2	3	66.6
Alexandria	-	4	4	100	2	2	4	50	-	-	-	-	-	-	-	-	-	-	-	-	-	-	-	-
Sharkia	-	2	2	100	5	3	8	37.5	3	2	5	40	2	1	3	33.3	-	-	-	-	-	3	3	100
Gharbia	-	3	3	100	7	3	10	30	4	-	4	0	-	4	4	100	-	-	-	-	4	-	4	0
Dakahlia	7	15	22	68.1	5	7	12	58	6	5	11	45.4	-	-	-	-	2	1	3	33.3	3	2	5	40
Qena	1	5	6	83	4	2	6	33.3	-	2	2	100	-	2	2	100	1	-	1	0	4	1	5	20
Total	38	42	80	52.5	29	23	52	44.2	17	15	32	46.8	3	13	17	76.4	6	3	9	33.3	15	9	24	43.5

N=Negative, P*=*Positive, T=Total, % +ve=Percent of positive

The results suggested that, because there was no history of vaccination, the detected antibodies were related to active infection. In the past few years, several outbreaks of PPR disease have been reported in Egypt [2,14,15]. These previous studies focused on animal markets, which play an essential role in disease transmission as there are no restrictions on animal movement during epidemics. The findings of high infection rates in our study confirmed the results in previous studies. Accordingly, the strategy applied to eradicate PPR disease is not effective, so there is a need for an innovative strategy to control and eliminate the disease based on a regular and effective vaccine against the disease among small ruminants, a test to distinguish between infected and vaccinated animals (DIVA), and controls placed on the transport of animals across borders.

The isolation of PPRV through cell culture techniques is considered the gold standard for accurately diagnosing infection, although it is time-consuming [16]. Vero cells are used for the propagation of PPRV as these cells are easily accessible and can easily be maintained and grown for a long time *in vitro* [17]. The results of the inoculated tissue culture for the samples with positive ELISA results (105) on Vero cells yielded 22 viral isolates as follows: Buffy coat (n=7), nasal swabs (n=4), ocular swabs (n=5), saliva swabs (n=3), and tissue samples (n=3). There were fewer yielded isolates than expected (22/105) (Table-4). This could be due to the nature of the virus as it is RNA virus, so it is heat-labile and any leakage in the cold chain in the transportation of samples and the cell culture used [17]. All negative samples were confirmed to be negative by RT-PCR. The positive tissue culture isolates were confirmed by molecular PCR analysis for the *PPRV F* gene. The products successfully amplified by PCR showed a 480 bp fragment from all 22 isolates.

**Table-4:** PPR tissue culture for the positive capture ELISA samples and RT-PCR analysis.

Governorates	Buffy coat			Nasal swab			Ocular swab			Saliva swab			Fecal swab			Tissues		
					
	T	CPE	RT-PCR	T	CPE	RT-PCR	T	CPE	RT-PCR	T	CPE	RT-PCR	T	CPE	RT-PCR	T	CPE	RT-PCR
Cairo (El Marg)	5	2	2	4	2	2	4	1	1	4	1	1	2	0	0	1	1	1
Giza	8	1	1	2	0	0	2	1	1	2	1	1	-	-	-	2	1	1
Alexandria	4	1	1	2	0	0	0	0	0	-	-	-	-	-	-	-	-	-
Sharkia	2	0	0	3	0	0	2	0	0	1	0	0	-	-	-	3	0	0
Gharbia	3	0	0	3	0	0	0	0	0	4	0	0	-	-	-	-	-	-
Dakahlia	15	2	2	7	1	1	5	2	2	-	-	-	1	0	0	2	1	1
Qena	5	1	2	2	1	1	2	1	1	2	1	1	-	-	-	1	0	0
Total	42	7	7	23	4	4	15	5	5	13	3	3	3	0	0	9	3	1

RT-PCR=Reverse transcription polymerase chain reaction, ELISA=Enzyme-linked immunosorbent assay, CPE=Cytopathic effect, T=Total

**Figure-2:**
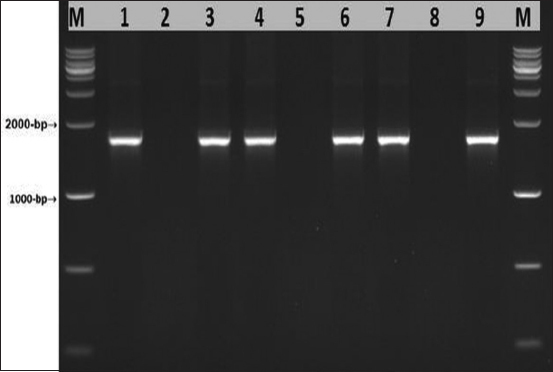
The polymerase chain reaction amplified of the peste des petits ruminants virus *F* gene full length (1641 bp). M lane M: DNA ladder. Lane: 1, 4, 5, and 8 negative samples. Lane: 2,3, 6, 7, and 9 positive samples.

**Figure-3:**
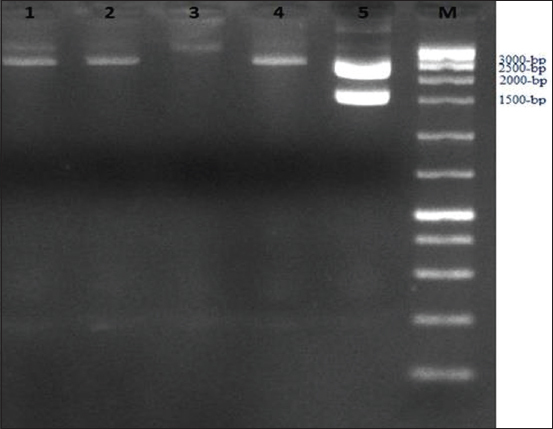
Agarose gel electrophoresis of digested recombinant vector by EcoRI Lane: 1-4 the undigested recombinant vectors. Lane M: DNA ladder (100, 200, 300, 400, 500, 700, 1000, 1500, 2000, 2500, 3000, 4000, 5000, 6000, 8000, and 10,000 bp). Lane: 5 is digested recombinant vector with two bands at 3000 bp (vector backbone) and 1641 bp (peste des petits ruminants virus *F* gene fragment).

The full length of the *PPRV F* gene is reported to be 1641 bp [14,18]. Tissue culture isolates confirmed to be positive by partial amplification of the *PPRV F* gene were used as templates for full-length amplification of the *PPRV F* gene. Figure-2 shows the successful amplification of the full-length *PPRV F* gene (1641 bp). The *PPRV F* gene cloning was performed to isolate the gene for several analyses. A molecular study of a positive recombinant vector inserted with the *PPRV F* gene was performed using a restriction enzyme and PCR-based method. The recombinant vector digested by *EcoRI* was run on an agarose gel to confirm the presence of the vector backbone and the inserted gene. The vector band was identified at 3000 bp, while the *PPRV F* gene band was found at 1641 bp (Figure-3).

**Figure-4:**
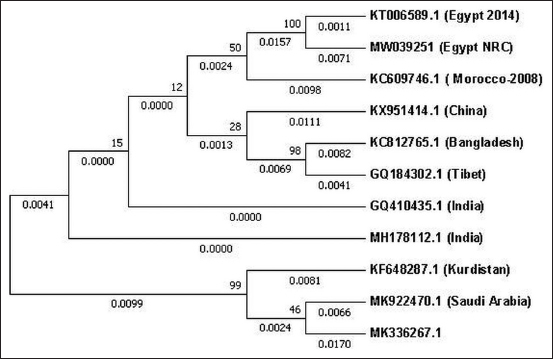
Phylogenetic tree showing the genetic relationships between isolates of peste des petits ruminants virus (MW039251 Egypt NRC) and the available accession numbers from GenBank. The tree was constructed based on peste des petits ruminants virus *F* gene sequences. The analysis was performed using the MEGA7 software [11] and the maximum likelihood method.

The sequence results of the products of the *PPRV F* gene successfully amplified by PCR were deposited in GenBank with accession numbers MW039251 and MW039252. Blast analysis of the sequence results showed 96.7% similarity with the PPRV fusion protein gene isolate from Egypt (KT006589.1, 2014) (Figure-4), 96.54% for one from Izatnagar/India (MH178112.1, 2018), 97% for one from Ethiopia (KJ867541.1, 2010), and 95.93% for a PPRV strain from Morocco (KC609746.1, 2013). The phylogenetic tree in Figure-4 shows the genetic relationships among the isolates of PPRV and the available accessions from GenBank. The phylogenetic analysis suggested that the circulating PPRV Egyptian strain belongs to the IV lineage [19,20].

Single-nucleotide polymorphism (SNP) is defined as a substitution of a single nucleotide at a specific position in the genome. SNPs in the coding region are of two types: Synonymous and non-synonymous SNPs. Synonymous SNPs do not affect the protein sequence, while non-synonymous SNPs change the amino acid sequence of the protein and can thus change the gene function [21].

**Figure-5:**
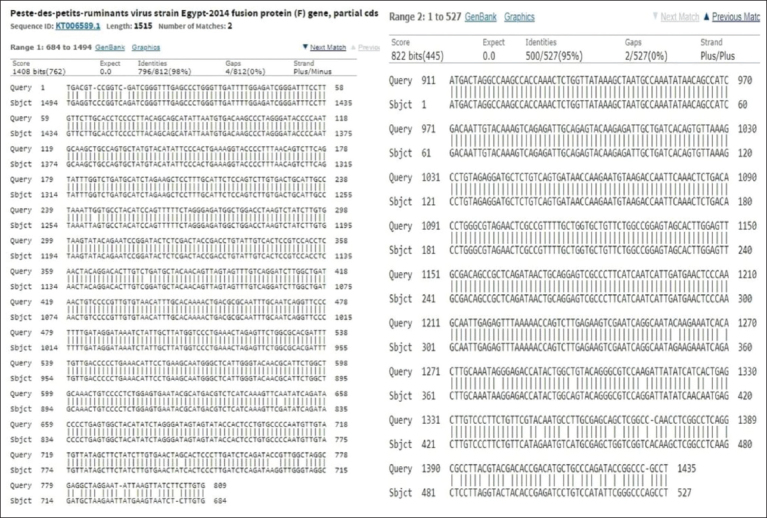
Blast analysis of the peste des petits ruminants virus *F* gene.

Blast analysis showed that the sequence of the studied *PPRV F* gene is 96.7% similar to the PPRV strain Egypt-2014 fusion protein (F) gene (sequence ID: KT006589.1) (Figure-5). They differ in terms of 43 SNPs. The obtained results suggested that this difference in 43 SNPs could play a role in the overall function of the *PPRV F* gene since the full length of the *PPRV F* gene encodes a specific protein responsible for virus virulence [4].

## Conclusion

The results of this study indicated that the emerging PPRV belongs to the IV lineage among small ruminant animals, which negatively affects animal production and the socioeconomic status of farmers. The findings also indicated the need for an innovative strategy to control and eliminate the disease based on a regularly administered and effective vaccine, a test to DIVA, and the need for further study on the protein structure and *PPRV F* gene expression, which will enable us to understand the molecular evolution of the virus and control and eliminate PPR disease.

## Authors’ Contributions

SA as the principal author, carried out the molecular laboratory work and wrote the manuscript. WAH and MM carried out the laboratory work related to ELISA and virus isolation. MAM performed the fieldwork, collected the samples, and drafted the manuscript. All authors read and approved the final manuscript.
